# Spleen-Derived Interleukin-10 Downregulates the Severity of High-Fat Diet-Induced Non-Alcoholic Fatty Pancreas Disease

**DOI:** 10.1371/journal.pone.0053154

**Published:** 2012-12-28

**Authors:** Koro Gotoh, Megumi Inoue, Kentaro Shiraishi, Takayuki Masaki, Seiichi Chiba, Kimihiko Mitsutomi, Takanobu Shimasaki, Hisae Ando, Kansuke Fujiwara, Isao Katsuragi, Tetsuya Kakuma, Masataka Seike, Toshiie Sakata, Hironobu Yoshimatsu

**Affiliations:** Department of Internal Medicine 1, Faculty of Medicine, Oita University, Yufu City, Oita, Japan; University of Valencia, Spain

## Abstract

Obesity is associated with systemic low-grade inflammation and is a risk factor for non-alcoholic fatty pancreas disease (NAFPD), but the molecular mechanisms of these associations are not clear. Interleukin (IL)-10, a potent anti-inflammatory cytokine, is released during acute pancreatitis and is known to limit inflammatory responses by downregulating the release of proinflammatory mediators. The origin of IL-10 that suppresses pancreatitis has not been investigated. Since obesity is known to reduce expression of proinflammatory cytokines in the spleen, we examined whether spleen-derived IL-10 regulates NAFPD caused by high-fat (HF) diet-induced obesity. The following investigations were performed: 1) IL-10 induction from spleen was examined in male mice fed a HF diet; 2) triglyceride content, expression of pro- and anti-inflammatory cytokines and infiltration of M1 and M2 macrophages were determined to evaluate ectopic fat accumulation and inflammatory responses in the pancreas of splenectomy (SPX)-treated mice fed HF diet; 3) exogenous IL-10 was systemically administered to SPX-treated obese mice and the resulting pathogenesis caused by SPX was assessed; and 4) IL-10 knockout (IL-10KO) mice were treated with SPX and ectopic fat deposition and inflammatory conditions in the pancreas were investigated. Obesity impaired the ability of the spleen to synthesize cytokines, including IL-10. SPX aggravated fat accumulation and inflammatory responses in the pancreas of HF diet-induced obese mice and these effects were inhibited by systemic administration of IL-10. Moreover, SPX had little effect on fat deposition and inflammatory responses in the pancreas of IL-10KO mice. Our findings indicate that obesity reduces IL-10 production by the spleen and that spleen-derived IL-10 may protect against the development of NAFPD.

## Introduction

Obesity leads to the infiltration of fat into multiple organs, including the liver, heart, kidneys, and pancreas. Furthermore, metabolic disorders associated with the obese state increase fatty acid flux to the liver and pancreas [1], [2]. Non-alcoholic fatty liver disease (NAFLD), a major form of chronic liver disease in adults and children, results from infiltration of fat into the liver [3]. Similar infiltration of the pancreas by fat (non-alcoholic fatty pancreatic disease [NAFPD]), increases the weight of the pancreas due to the accumulation of high levels of triglycerides (TGs) and free fatty acids (FFAs), which can compromise normal pancreatic function. Pancreatic fat accumulation is also characterized by increased production of cytokines that are released locally and may result in inflammation, which is associated with organ dysfunction [2]. NAFPD has been shown to be involved in pancreatic fibrogenic processes, through the necrosis-fibrosis sequence and via a direct inflammatory pathway [4]. Pancreatic stellate cells (PSC), which are similar to hepatic stellate cells, play a major role in this process [5]. Interleukin (IL)-10, a potent anti-inflammatory cytokine, was reported to be released during the course of experimental acute pancreatitis and was shown to limit the severity of this disease by downregulating the release of proinflammatory mediators [6], [7]. IL-10 has also been reported to have direct antiproliferative and antifibrotic effects [8], [9].

The spleen is the largest lymphoid organ in the body and plays an important role in host immune function. In one study, obese rats exhibited decreased expression of genes encoding pro-inflammatory cytokines such as IL-6 and tumor necrosis factor α (TNF-α) in the spleen [10]. In contrast, IL-10, which is synthesized by several cell types in multiple organs, including the spleen, inhibits the synthesis of pro-inflammatory cytokines. New evidence indicates that activated B-cells, which mature in the marginal zone of the spleen, produce large amounts of IL-10 and play a regulatory role in suppressing harmful immune responses [11]. Indeed, low IL-10 production has been demonstrated in obesity [12], [13].

Based on the evidence in the literature, we hypothesize that obesity suppresses the synthesis of IL-10 and thereby results in chronic inflammation in the pancreas. The data from the present study demonstrate that obesity reduces IL-10 expression in the spleen, and that spleen-derived IL-10 protects against obesity-induced inflammatory responses in the pancreas.

## Research Design and Methods

### Animals

Male C57Bl/6J mice (wild-type mice, 22–25 g; KBT Oriental, Japan) and IL-10-deficient mice (IL-10KO mice, 002251-B6.129P2-*Il10^tm1Cgn^*/J, a gift from Sandy Morse, The Jackson Laboratory, Bar Harbor, ME) were housed in a room at Oita University under a 12/12-h light/dark cycle with lights on from 07∶00 to 19∶00. IL-10KO mice, maintained at our university, were used for backcrossing. The following polymerase chain reaction (PCR) primers were used for genotyping: 5′-CCACACGCGTCACCTTAATA-3′ (mutant forward), 5′-GTTATTGTCTTCCCGGCTGT-3′ (wild-type reverse), and 5′-CTTGCACTACCAAAGCCACA-3′ (common). All studies were conducted in accordance with guidelines of Oita University, based on the Guide for the Care and Use of Laboratory Animals published by the US National Institutes of Health. Additionally, this study was specifically approved by the ethics committee of the Division of Laboratory Animal Science, Research Promotion Project of Oita University.

### Surgery Procedure

The mice were anesthetized by intraperitoneal (i.p.) injection of sodium pentobarbital (100 mg/kg). To perform the splenectomy (SPX), the abdominal cavity was opened, the two major sources of blood flow (splenic artery and gastric artery) were tied off with sutures proximal to the spleen and ligated, and the spleen was carefully removed [14]. SPX was performed in 15 min. For the sham operation, the abdomen was opened, but the spleen was not removed. For all surgical procedures, the abdomen was closed in one layer and 200 µl of normal saline was administered subcutaneously. No mice died as a result of the procedure.

### Experimental Protocol

#### Experiment 1

Wild-type mice were assigned to one of two groups (*n* = 6 in each group) as follows: group 1 mice were fed standard chow (Standard: 10% fat, 70% carbohydrate, 20% protein; Diet Research, New Brunswick, NJ) for 8 weeks and then subjected to the sham operation; group 2 mice were fed a high-fat diet (HF: 60% fat, 20% carbohydrate, 20% protein; Diet Research) for 8 weeks and then underwent the sham operation.

#### Experiment 2

Wild-type mice were assigned to one of two groups (*n* = 6 in each group) as follows: group 1 mice were fed Standard for 8 weeks and then subjected to the sham operation; group 2 mice were fed Standard for 8 weeks and then underwent SPX.

#### Experiment 3

Wild-type mice were assigned to one of five groups (*n* = 6 in each group) as follows: group 1 mice were fed Standard for 8 weeks after the sham operation and administered mouse serum albumin (m-albumin); group 2 mice were fed HF for 8 weeks after the sham operation and then given m-albumin; group 3 mice were fed HF for 8 weeks after SPX and then given m-albumin; group 4 mice were fed HF for 8 weeks after SPX and then given recombinant mouse IL-10 (r-IL-10, 0.5 ng/day; Wako Chemicals, Osaka, Japan); group 5 (pair-fed group) mice were fed the amount of food consumed by the SPX-treated group for 8 weeks after the sham operation and then given m-albumin.

#### Experiment 4

Wild-type and IL-10KO mice were assigned to one of three groups (*n* = 6 in each group) as follows: group 1 mice were fed HF for 8 weeks after the sham operation and administered m-albumin; group 2 mice were fed HF for 8 weeks after SPX and administered m-albumin; and group 3 mice were fed HF for 8 weeks after SPX and administered r-IL-10 (0.5 ng/day; Wako Chemicals).

Osmotic pumps (Durect Corp., Cupertino, CA, USA) containing m-albumin or r-IL-10 were implanted into the backs of all mice, parallel to the spine, for 4 weeks. The dose of recombinant mouse IL-10 was determined by multiplying the average normal serum concentration of IL-10 (24 pg/ml) by the average total blood volume of a 40-g mouse. Food intake over 24 h was calculated by weighing the remaining food, and body weight was determined between 17∶00 and 18∶00 every day. Food intake was normalized according to body weight. All mice were housed for an additional 4 weeks after completion of the interventions. All mice were anesthetized with sodium pentobarbital and exsanguinated following transcardiac perfusion with 100 ml of saline containing 200 units of heparin. The spleen and pancreas were removed and cleaned to remove peritoneal fat and lymph nodes.

### Cytokine Levels in the Spleen, Pancreas, and Serum

Commercially available enzyme-linked immunosorbent assay (ELISA) kits (Invitrogen, Carlsbad, CA, USA) were used to measure IL-1β, monocyte chemotactic protein-1 (MCP-1), and IL-10 levels in the spleen, pancreas, and serum. The concentration of protein in each organ was determined by the Lowry method. IL-10/IL-1β ratios were also calculated.

### Histological and Immunohistochemical Analyses

Pancreas samples were fixed in 4% buffered paraformaldehyde, embedded in paraffin, sectioned, deparaffinized in xylene and stained with Mayer’s hematoxylin and eosin (H&E) (Wako Chemicals), Mallory-Azan reagent to assess fibrosis and with oil-Red-O staining to evaluate the distribution of fat.

For immunohistochemical staining of CD11c, CD206 and insulin, 5-µm-thick frozen pancreas sections were incubated overnight at 4°C with rabbit anti-mouse CD11c (Novus Biochemical, Littleton, CO, USA), rat anti-mouse CD206 (AbD Serotec, Oxford, UK), rabbit anti-mouse insulin (Acris Antibodies GmbH, Herford, Germany) and α-smooth muscle actin (α-SMA; Abcam, Cambridge, MA, USA). Slides were subsequently washed with phosphate-buffered saline (PBS) and incubated with biotin-conjugated goat anti-rabbit or rabbit anti-rat IgG (ABC reagent; Vector Laboratories, Burlingame, CA, USA). The immunoreactivity of each sample was visualized with DAB (Nacalai Tesque, Kyoto, Japan). Normal rabbit or rat serum was used in place of the aforementioned antibodies as a negative control and yielded no staining. The percentage of area immunostained for CD11c and CD206 in five portions of pancreas per animal was analyzed using Mac Scope version 6.02 (Mitani Shoji, Fukui, Japan). M1/M2 was calculated as CD11c/CD206. Insulin areas were quantified in insulin-stained slides. Twenty islets per animal were analyzed. All islets in the slide were circled, and the insulin-stained area (mm^2^) for each islet was quantified using Mac Scope version 6.02. α-SMA staining was quantified by counting the number of α-SMA-positive cells in five high-power fields (HPF, ×400 magnification) per section. Positive cells located near blood vessel walls were ignored. Data were expressed as the number of α-SMA-positive cells per 50 acinar cells to correct for the variance in the number of cells per HPF among all groups. Quantification was performed by two investigators who had no knowledge of the identity of the experimental groups.

### Pancreatic Fibrosis

Pancreas sections were subjected to Mallory-Azan staining. The mean fibrotic area in 20 islets per animal, identified as blue coloration, was evaluated as a percentage of the field using Mac Scope version 6.02. Hydroxyproline, a marker of total collagen content in the pancreas, was measured with a hydroxyproline ELISA kit (USCN Life Science, Inc., Houston, TX, USA).

### Pancreatic Triglyceride (TG) Content and Fasting Blood Glucose and Serum Insulin, TG, Free Fatty Acid (FFA), Total Cholesterol (TC) and Leptin Levels

After an overnight fast, blood glucose concentrations were measured using the glucose oxidase method and a glucose analyzer (MS-GR101; Terumo, Tokyo, Japan). Serum insulin and leptin concentrations were determined using insulin and leptin ELISA kits (Shibayagi, Gunma, Japan). The TG content of pancreas samples, and serum TG, FFA, and TC concentrations were determined using commercially available kits (Wako Chemicals).

### Statistics

Results were expressed as mean±SEM. Statistical tests included 2-tailed Student’s *t* test and 2-way ANOVA followed by Scheffe’s test for *post hoc* comparison. For all tests, the level of significance was set at *p*<0.05.

## Results

### HF Feeding Reduces Serum Levels of IL-10, but not IL-1β or MCP-1

Although splenic expression of IL-1β, MCP-1, and IL-10 was significantly lower in mice in the HF diet group compared to those in the standard chow group, in serum, only IL-10, but not IL-1β or MCP-1, was significantly lower in the HF diet group compared to the standard chow group (Table 1). Serum cytokine levels, with the exception of IL-10, are likely maintained by organs other than the spleen when splenic cytokine expression is downregulated following consumption of a HF diet. Serum IL-10 levels remained low, however, suggesting that large amounts of IL-10 in the serum are derived from the spleen.

**Table 1:** Effects of a HF diet on splenic and serum levels of pro- and anti-inflammatory cytokines.

	IL-β	MCP-1	IL-10
Spleen (pg/mg protein)			
Standard	39.8 ± 4.2	19.7 ± 1.2	21.5 ± 1.5
HF	21.4 ± 3.1 *	10.3 ± 1.4 *	8.8 ± 1.1 *
Serum (pg/ml)			
Standard	13.7 ± 3.4	18.6 ± 3.1	30.5 ± 4.8
HF	15.1 ± 2.6	19.4 ± 2.4	18.5 ± 2.7 *

Protein levels of IL-1β, MCP-1, and IL-10 in the spleen and serum from each group (*n* = 6). *P<0.05 vs. the Standard group. Treatment groups: Standard, fed standard chow and sham-operated; HF, fed a HF diet and sham-operated.

### SPX Causes Hypophagia, Body Weight Loss, Elevation of TG and FFA Levels, and Reduction of Serum Adiponectin Level

We investigated whether splenectomy contributes to inflammation in the pancreas. First, we examined the effect of splenectomy on energy metabolism. SPX resulted in lower energy intake, body weight, weight of epididymal white adipose tissue (WAT), and interestingly lower serum adiponectin levels compared to sham-operated mice (Table 2). There were, however, no significant differences in fasting blood glucose and serum insulin levels between the two groups. In addition, SPX also resulted in increased serum levels of TG and FFA, but not TC, compared with the sham treatment (Table 2).

**Table 2:** Effects of splenectomy on daily food intake, body weight, epididymal WAT weight, serum adiponectin levels, glucose and lipid metabolism.

	Standard-Sham	Standard-SPX
Daily food intake (cal/g weight)	182.8±14.8	148.3±10.7 *
Body weight (g)	48.4±3.2	40.3±2.2 *
Epididymal WAT (g)	13.8±1.16	10.8±1.11 *
Serum adiponectin level(µg/ml)	7.54±0.46	5.13±0.57 *
Blood glucose level (mg/dl)	89.2±3.5	91.1±2.6
Serum insulin level (ng/ml)	1.45±0.16	1.38±0.22
Serum TG level (mg/dl)	39.3±2.6	48.6±10.5 *
Serum FFA level (mEq/l)	178.1±14.4	208.3±10.5 *
Serum TC level (mg/dl)	56. 2±8.2	58.8±13.1

* *P*<0.05 vs. the Standard-Sham group. Treatment groups: Standard-Sham, fed standard chow and sham-operated (*n* = 6); Standard-SPX, fed standard chow and splenectomized (*n* = 6).

### SPX Enlarges the Insulin-positive Area and Accelerates Fibrosis and Lipid Accumulation

**Figure 1:**
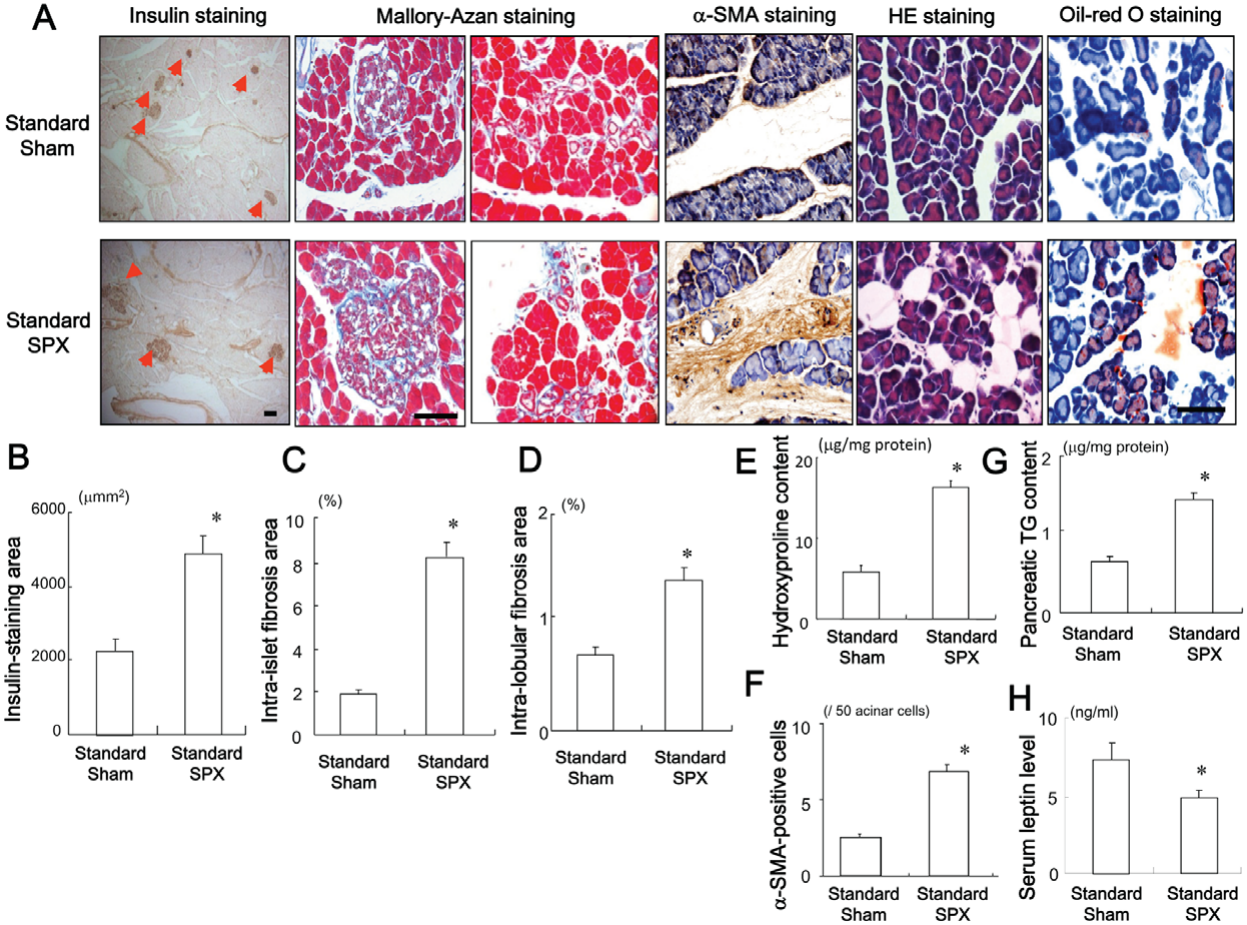
Effects of SPX on islets and fat accumulation in the pancreas. (**A**) Representative images of insulin staining (left), Mallory-Azan staining (middle), and H&E staining (right) of pancreas sections from mice in each group. (**B−E**) Insulin staining area (**B**), intra-islet fibrosis area (**C**), intra-lobular fibrosis area (**D**), hydroxyproline content (**E**), α-SMA-positive cells (**F**), TG content (**G**) in the pancreas and serum leptin level (**H**) in each group (*n* = 6). *P<0.05 vs. the Standard-Sham group. Scale bar = 100 µm. Treatment groups: Standard-Sham, fed standard chow and sham-operated; Standard-SPX, fed standard chow and splenectomized.

The effects of SPX on the insulin-stained area, intra-islet and intra-lobular fibrosis, and fat accumulation in the pancreas were evaluated. SPX increased the insulin-stained area and disrupted the islet architecture and islet boundaries compared to sham treatment (Figure 1A and 1B). SPX also aggravated intra-islet (Figure 1A and 1C) and intra-lobular fibrosis (Figure 1A and 1D), increased hydroxyproline content (Figure 1E) and the percentage of α-SMA-positive cells (Figure 1A and 1F), elevated TG content and fat accumulation in the pancreas (Figure 1A and 1G) and led to elevated serum leptin levels (Figure 1H) compared to sham treatment. Results from H&E and oil-red O staining showed that SPX increased fat deposition mainly in intra-lobular areas but not in intra-acinar cell areas, whereas small vacuoles within acinar cells were positive for oil-red O staining (Figure 1A).

### SPX Promotes Infiltration of M1 and M2 Macrophages in the Pancreas

**Figure 2:**
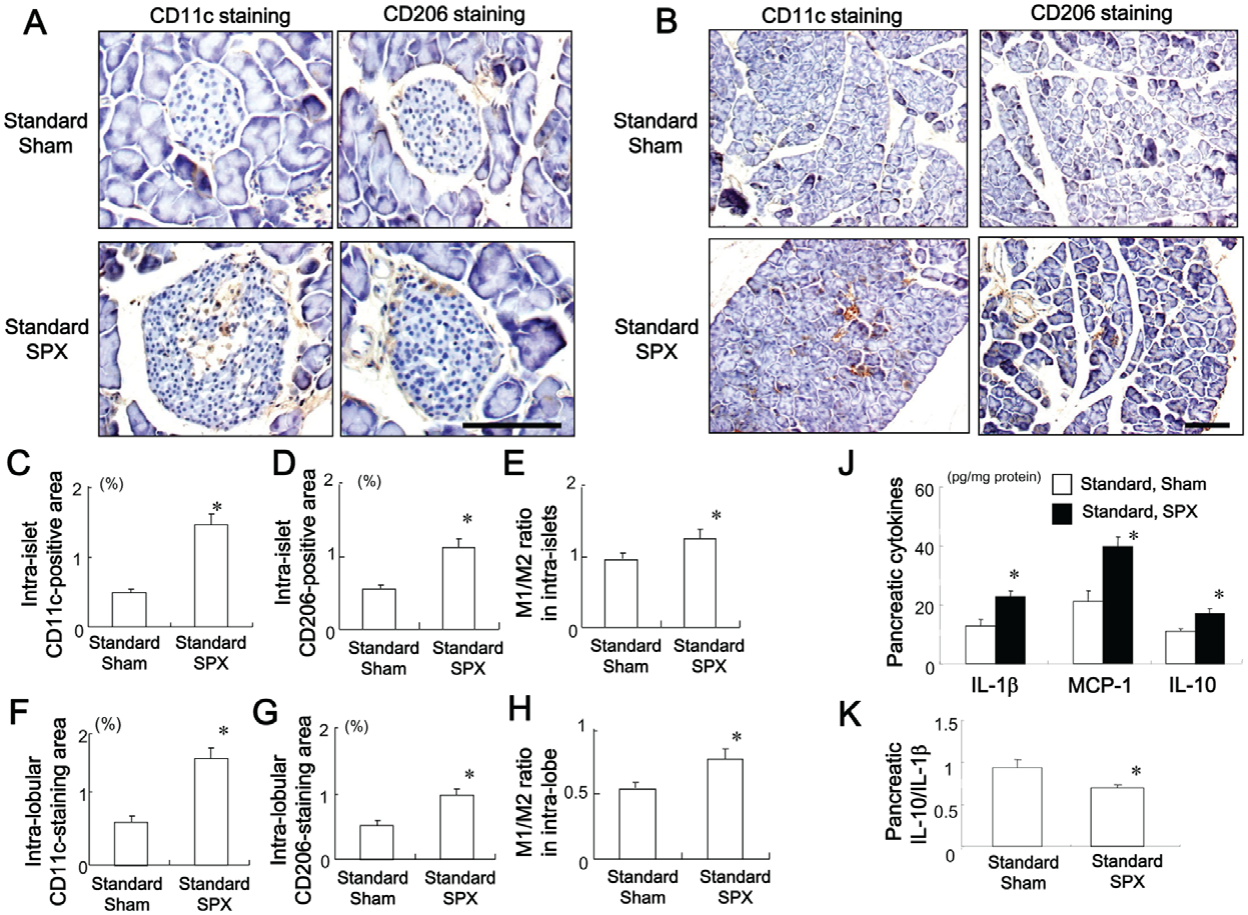
Effects of SPX on the infiltration of M1 and M2 macrophages and on inflammatory responses in the pancreas. (**A and B**) Representative images of CD11c staining (left) and CD206 staining (right) in intra-islet areas (**A**) and intra-lobular areas (**B**) in pancreas sections from each group. (**C−E**) Percentage of CD11c-positive area (**C**) and CD206-positive area (**D**) and M1/M2 ratio (**E**) in intra-islet areas. (**F−H**) Percentage of CD11c-positive area (**F**) and CD206-positive area (**G**) and M1/M2 ratio (**H**) in intra-lobular areas. (**J and K**) Content of pro- and anti-inflammatory cytokines (**J**) and interleukin (IL)-10/IL-1β ratio (**K**) in the pancreas in each group (*n* = 6). *P<0.05 vs. the Standard-Sham group. Scale bar = 100 µm. Treatment groups: Standard-Sham, fed standard chow and sham-operated; Standard-SPX, fed standard chow and splenectomized.

The effects of SPX on the pro-inflammatory response in the pancreas were evaluated. There was marked infiltration of CD11c (M1 macrophage marker)-positive and CD206 (M2 macrophage marker)-positive cells into pancreatic intra-islet (Figure 2A, 2C, 2D) and intra-lobular (Figure 2B, 2F, 2G) areas in SPX-treated mice. We then assessed the CD11c/CD206-positive cell ratio as a measure of macrophage polarization in the two groups. Interestingly, the CD11c/CD206 ratio in both islet and lobular areas was significantly higher in SPX mice as compared with sham-operated mice, which may reflect a shift in the macrophages toward a pro-inflammatory state. We defined the CD11c/CD206 ratio as the M1/M2 ratio (Figure 2H). Moreover, SPX increased pro- and anti-inflammatory cytokine levels (Figure 2J) in the pancreas, but reduced the pancreatic IL-10/IL-β ratio (Figure 2K). This is consistent with a previous finding that the IL-10/TNF-α ratio is low in non-alcoholic steatohepatitis (NASH) [12].

### IL-10 Treatment Inhibits SPX-induced Alterations in the Pancreas

**Figure 3:**
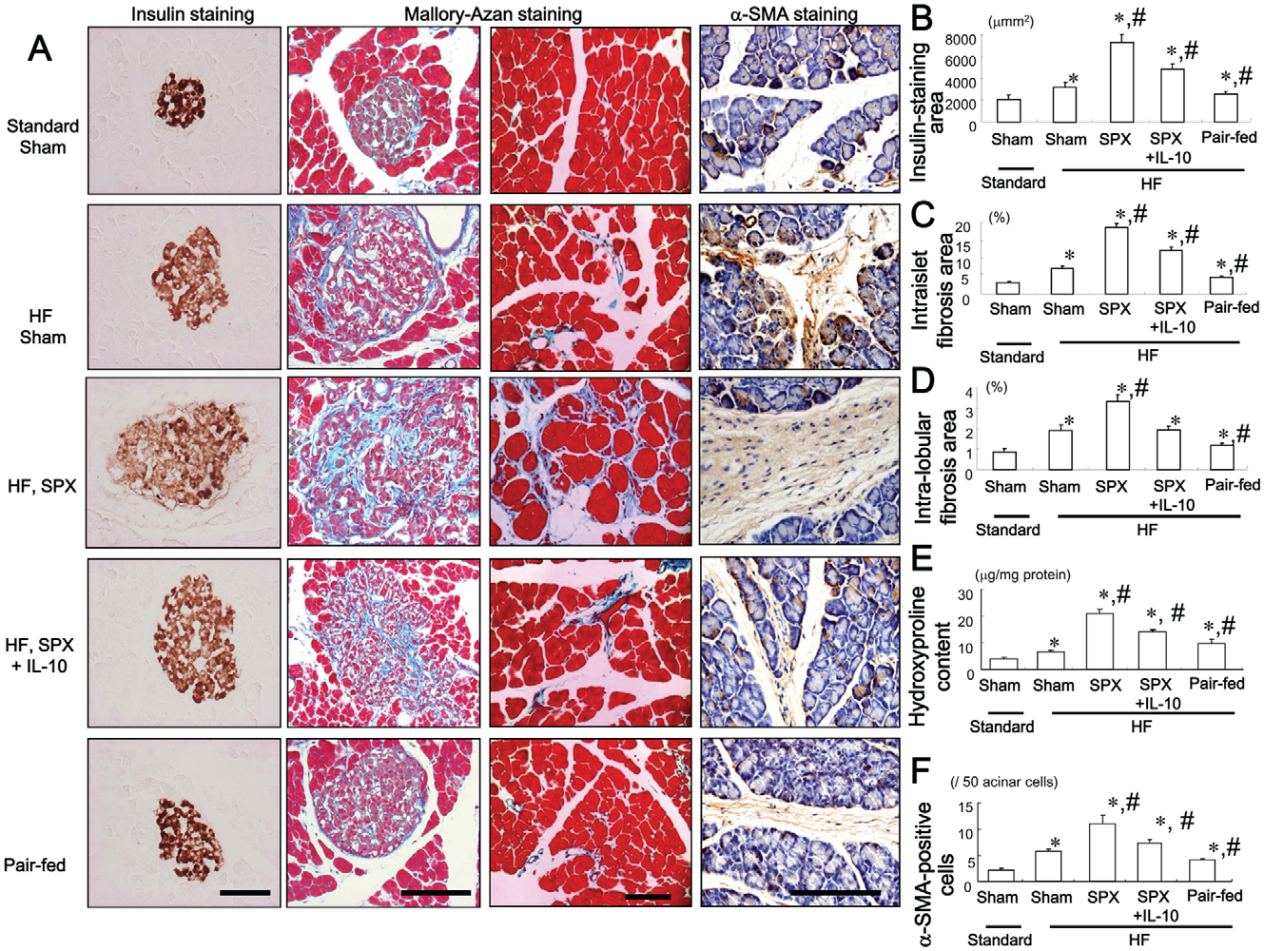
Systemic administration of IL-10 diminishes SPX-induced alterations in pancreatic islets. (**A**) Representative images of insulin staining (left) and Mallory-Azan staining (right) in pancreas sections from each group. Scale bar = 100 µm. (**B−F**) Insulin-staining area (**B**), intra-islet (**C**) and intra-lobular (**D**) fibrosis area, hydroxyproline content (**E**) and α-SMA-positive cells (**F**) in the pancreas in each group (*n* = 6). *P<0.05 vs. the Sham-Standard group, ^#^ *P*<0.05 vs. the Sham-HF group. Treatment groups: Standard-Sham, fed standard chow, given serum albumin, and sham-operated; HF-Sham, fed a HF diet, administered serum albumin, and sham-operated; HF-SPX, fed a HF diet, given serum albumin, and splenectomized; HF-SPX+IL-10, fed a HF diet, given IL-10, and splenectomized; Pair-fed, administered serum albumin, sham-operated, and fed the same amount of food as that consumed by the HF-SPX group.

The effect of IL-10 on SPX-induced alterations in the islets was evaluated. Consumption of the HF diet increased insulin-stained areas (Figure 3A and 3B), islet hypertrophy and intra-islet fibrosis (Figure 3A and 3C), intra-lobular fibrosis (Figure 3A and 3D), pancreatic hydroxyproline content (Figure 3E) and the number of α-SMA-positive cells (Figure 3A and 3F) compared to the standard chow diet. SPX accelerated the islet hypertrophy, intra-islet and intra-lobular fibrosis induced by the HF diet (Figure 3). These SPX-induced changes were partially reversed, however, by IL-10 treatment (Figure 3). On the other hand, pair-feeding led to a decrease in the insulin-stained area, intra-islet and intra-lobular fibrosis compared to HF diet-fed sham mice (Figure 3).

### IL-10 Treatment Suppresses SPX-induced Infiltration of M1 and M2 Macrophages in the Intra-islet Area

**Figure 4:**
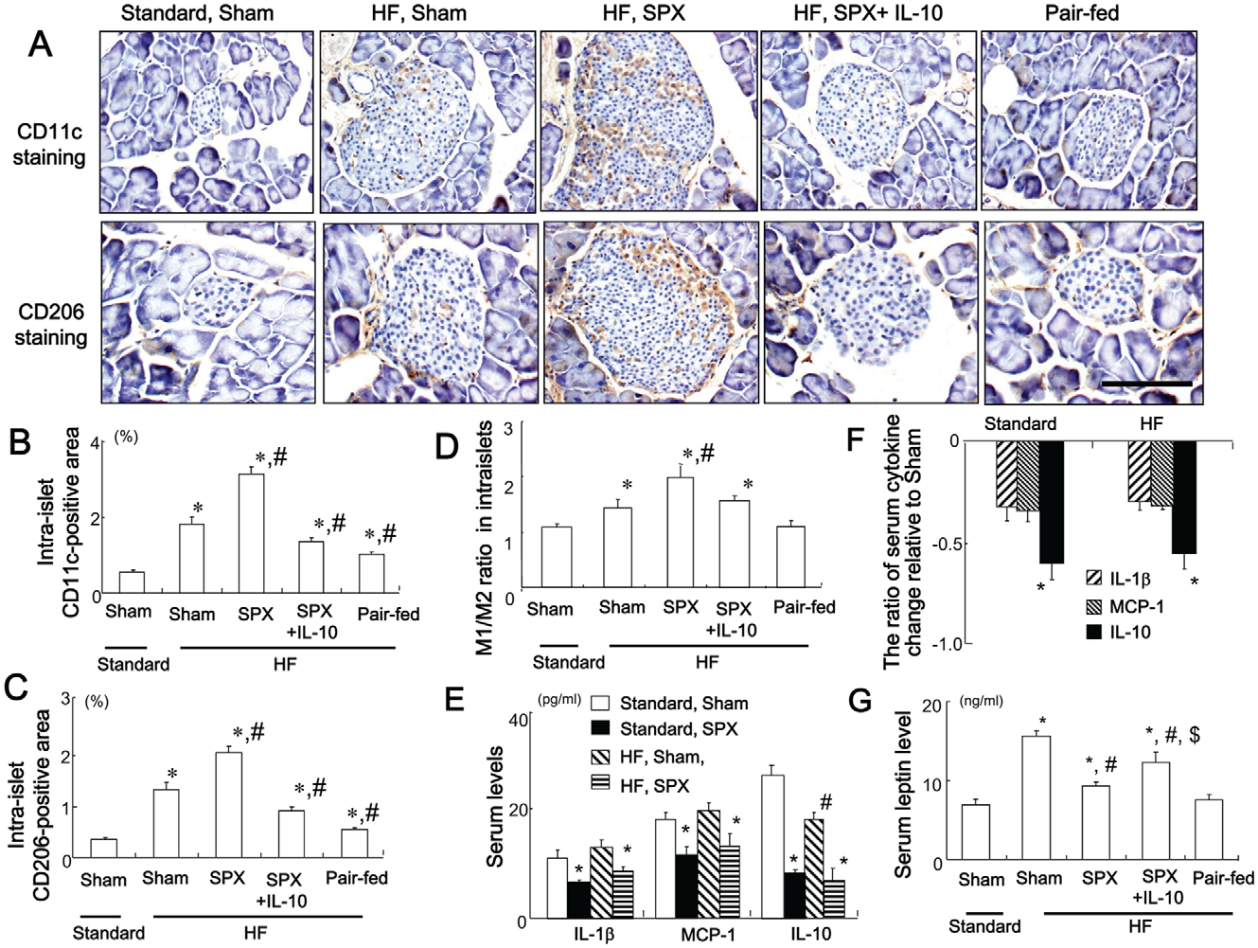
Systemic administration of IL-10 diminishes SPX-induced fat accumulation, infiltration of macrophages, and pro-inflammatory responses in the islets. (**A**) Representative images of CD11c staining (upper sections) and CD206 staining (lower sections) in intra-islet areas in pancreas sections from each group. Scale bar = 100 µm. (**B−D**) CD11c-positive areas (**B**), CD206-positive areas (**C**), and M1/M2 ratios (**D**) in the islets in each group (*n* = 6). *P<0.05 vs. the Standard-Sham group, ^#^ *P*<0.05 vs. the HF-Sham group. (**E**) Serum levels of IL-1β, MCP-1 and IL-10 in sham and SPX mice fed standard chow or a HF diet. *P<0.05 vs. the Standard-Sham group, ^#^ *P*<0.05 vs. the Standard-SPX group. (**F**) Serum cytokine changes relative to the Standard-Sham group. *P<0.05 vs. IL-1β and MCP-1. (**G**) Serum leptin levels in each group (n = 6). *P<0.05 vs. the Standard-Sham group, ^#^ *P*<0.05 vs. the HF-Sham group, ^$^ *P*<0.05 vs. the HF-SPX group. Treatment groups: Standard-Sham, fed standard chow, given serum albumin, and sham-operated; HF-Sham, fed a HF diet, administered serum albumin, and sham-operated; HF-SPX, fed a HF diet, given serum albumin, and splenectomized; HF-SPX+IL-10, fed a HF diet, given IL-10, and splenectomized; Pair-fed, administered serum albumin, sham-operated, and fed the same amount of food as that consumed by the HF-SPX group.

We examined whether SPX promoted HF diet-induced inflammation in the pancreas. The HF diet increased infiltration of CD11c-positive cells (M1 macrophages) (Figure 4A and 4B) and CD206-positive cells (M2 macrophages) (Figure 4A and 4C), as well as the M1/M2 ratio (Figure 4D) in the intra-islet area compared to standard chow. SPX further increased the infiltration of CD11c-positive and CD206-positive cells and the M1/M2 ratio in the intra-islet area in HF diet-fed mice (Figure 4A−D) and this was suppressed by IL-10 treatment (Figure 4A−D), suggesting that IL-10 may ameliorate SPX-induced inflammation in the pancreas. In addition, pair-feeding reduced these changes in macrophage infiltration and the M1/M2 ratio in the pancreas as compared to HF diet-fed sham-operated mice (Figure 4A−D).

To clarify whether spleen-derived IL-10 plays a greater role in inflammation than IL-10 produced locally in the pancreas, we examined serum cytokine levels in sham and SPX mice fed standard chow or the HF diet. SPX resulted in reduced serum pro- and anti-inflammatory cytokine levels, irrespective of diet (Figure 4E). Specifically, serum IL-10 levels were reduced by approximately 60% in mice fed the standard chow and HF diets who underwent SPX, which is a greater reduction than that observed for the other pro-inflammatory cytokines examined (Figure 4F). Interestingly, SPX diminished the HF-induced elevation of serum leptin level but led to increased fat accumulation in the pancreas (Figure 4G). Additionally, this attenuation was weakened by systemic IL-10 administration, supporting our previous findings that SPX decreased accumulation of visceral fat and IL-10 administration facilitated this accumulation [15].

### IL-10 Treatment Suppresses SPX-induced Fat Accumulation and Inflammatory Responses in the Pancreas

**Figure 5:**
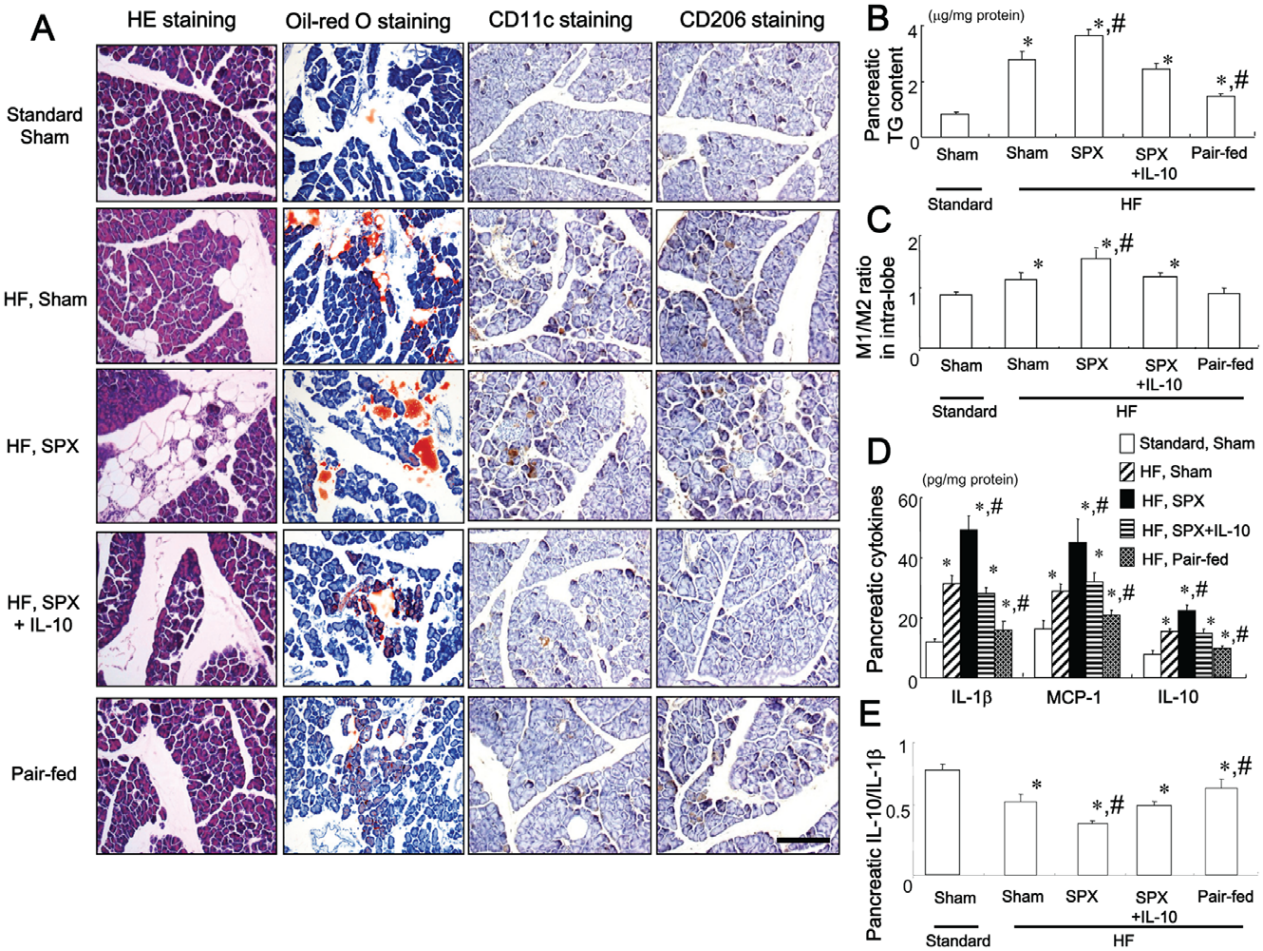
Systemic administration of IL-10 diminishes SPX-induced fat accumulation, infiltration of macrophages, and pro-inflammatory responses in the pancreas. (**A**) Representative images of H&E staining, oil-red O staining, CD11c staining, and CD206 staining in intra-lobular areas in pancreas sections from each group. Scale bar = 100 µm. (**B−E**) TG content (**B**), M1/M2 ratios (**C**), content of pro- and anti-inflammatory cytokines (**D**), and IL-10/IL-1β ratios (**E**) in the pancreas of each group (*n* = 6). *P<0.05 vs. the Standard-Sham group, ^#^ *P*<0.05 vs. the HF-Sham group. Treatment groups: Standard-Sham, fed standard chow, given serum albumin, and sham-operated; HF-Sham, fed a HF diet, administered serum albumin, and sham-operated; HF-SPX, fed a HF diet, given serum albumin, and splenectomized; HF-SPX+IL-10, fed a HF diet, given IL-10, and splenectomized; Pair-fed, administered serum albumin, sham-operated, and fed the same amount of food as that consumed by the HF-SPX group.

As shown by morphological findings (Figure 5A) and analysis of pancreatic TG content (Figure 5B), consumption of the HF diet increased fat accumulation in the intra-lobular areas but not in the intra-acinar cells areas of the pancreas, and increased pancreatic TG content compared to the standard chow. HF diet-fed SPX mice showed increased fat accumulation compared to HF diet-fed sham-operated mice, despite concurrent hypophagia and body weight loss. These SPX-derived changes were suppressed by IL-10 treatment (Figure 5A and 5B). In contrast, pair-feeding resulted in reduced accumulation of fat in the intra-lobular areas of the pancreas as compared with HF diet-fed sham-operated mice (Figure 5A and 5B), suggesting that the fat accumulation may have been caused by factors other than nutrient excess.

We further investigated whether the inflammatory response induced by SPX in the intra-lobular area of the pancreas was similar to that in the intra-islet area. We found that the HF diet increased the M1/M2 ratio, and pro- and anti-inflammatory cytokine levels (Figure 5A, 5C, 5D) but decreased the IL-10/IL-1β ratio (Figure 5E) in the pancreas compared to the standard chow diet. SPX further increased the M1/M2 ratio, and levels of pro- and anti-inflammatory cytokines, and reduced the IL-10/IL-1β ratio in the pancreas of HF diet-fed mice (Figure 5A, 5C−E), indicating that SPX induces an inflammatory response. IL-10 treatment improved SPX-induced inflammatory responses in the pancreas (Figure 5A, 5C−E). Pair-feeding on the other hand, reduced the M1/M2 ratio, pro- and anti-inflammatory cytokine levels and elevated the IL-10/IL-1β ratio in the pancreas compared to HF diet-fed sham-operated mice (Figure 5A, 5C−E).

### SPX has Little Effect on Islet Hypertrophy, Fibrosis or the Pancreatic Inflammatory Response in IL-10-deficient (IL-10KO) Mice

**Figure 6:**
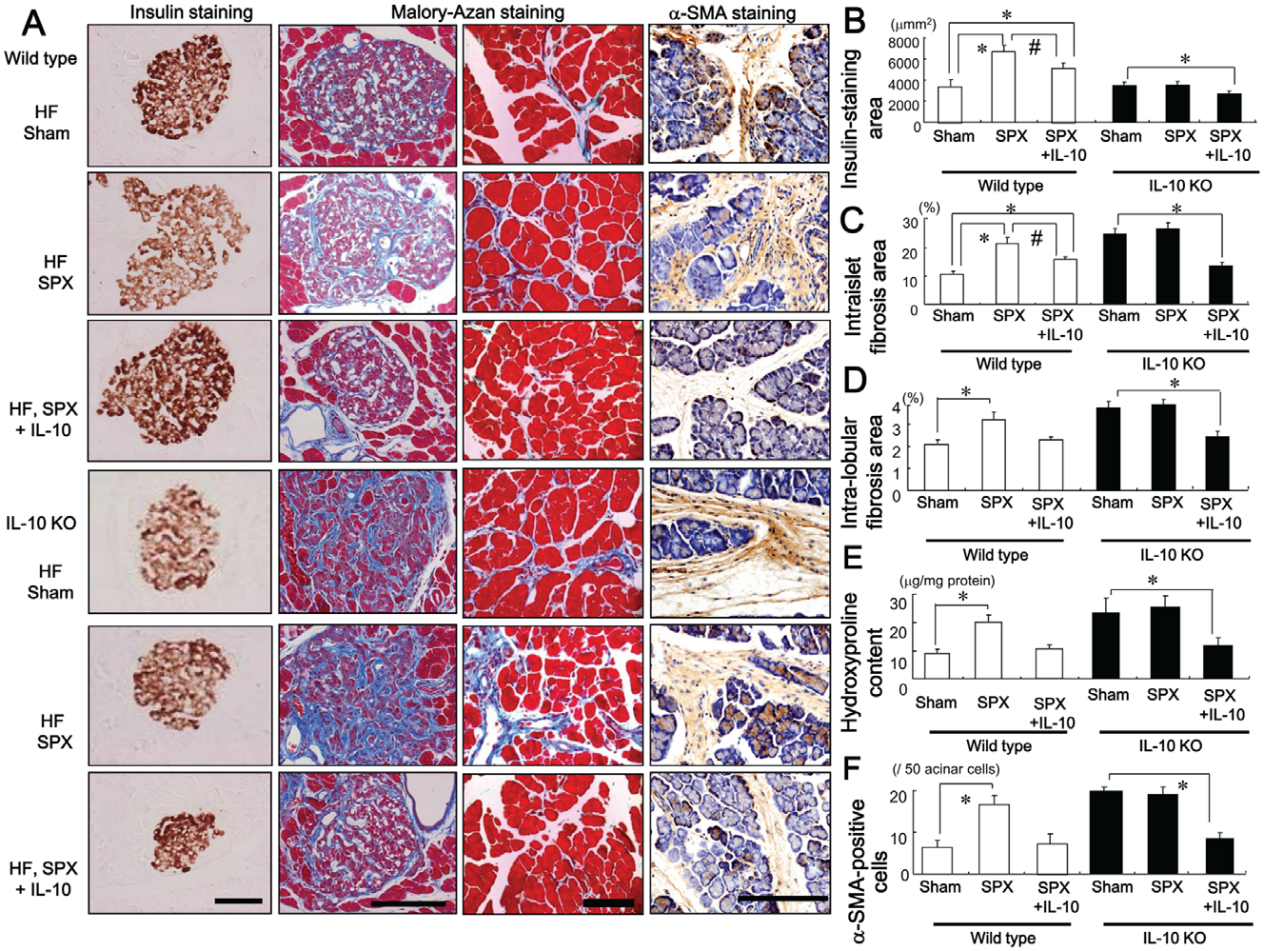
SPX has little effect on changes in the pancreatic islets in IL-10-deficient mice. (**A**) Representative images of insulin staining (left), Mallory-Azan staining (middle), and α-SMA staining (right) in pancreas sections from each group. Scale bar = 100 µm. (**B−F**) Insulin-staining area in the pancreas (**B**), intra-islet fibrosis area (**C**) and intra-lobular fibrosis area (**D**), hydroxyproline content (**E**) and α-SMA-positive cells (**F**) in each group (*n* = 6). *P<0.05 vs. the Sham group (wild-type or IL-10KO mice), ^#^ *P*<0.05 vs. SPX mice (wild-type). Treatment groups: Sham, fed a HF diet, given serum albumin and sham-operated; SPX, fed a HF diet, given serum albumin, and splenectomized; SPX+IL-10, fed a HF diet, given recombinant mouse IL-10 and splenectomized. Wild-type, wild-type mice; IL-10KO, IL-10-deficient mice.

**Figure 7:**
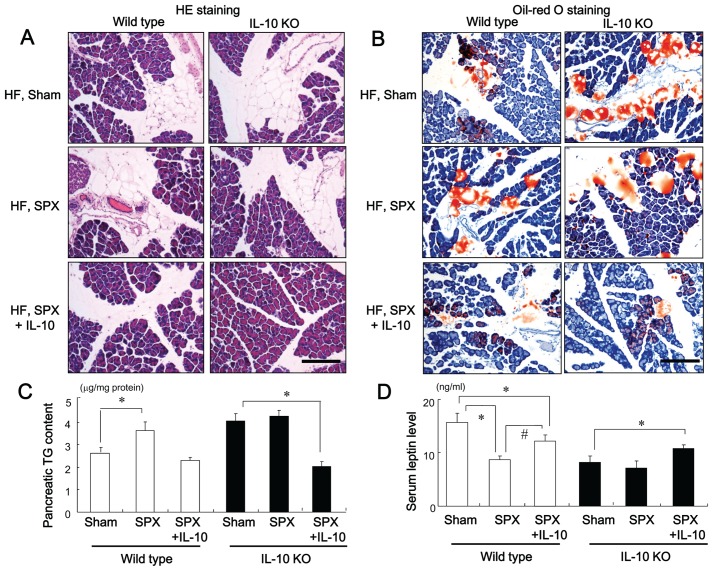
SPX has little effect on fat accumulation in the pancreas in IL-10-deficient mice. (**A** and **B**) Representative H&E staining (**A**) and oil-red O staining (**B**) in intra-lobular areas in pancreas sections from each group. Scale bar = 150 µm. (**C** and **D**) Pancreatic TG content (**C**) and serum leptin level (**D**) in each group (*n* = 6). *P<0.05 vs. the Sham group (wild-type or IL-10KO mice), ^#^ *P*<0.05 vs. SPX mice (wild-type). Treatment groups: Sham, fed a high-fat (HF) diet, given serum albumin and sham-operated; SPX, fed a HF diet, given serum albumin, and splenectomized; SPX+IL-10, fed a HF diet, given recombinant mouse IL-10, and splenectomized. Wild-type, wild-type mice; IL-10KO, IL-10-deficient mice.

**Figure 8:**
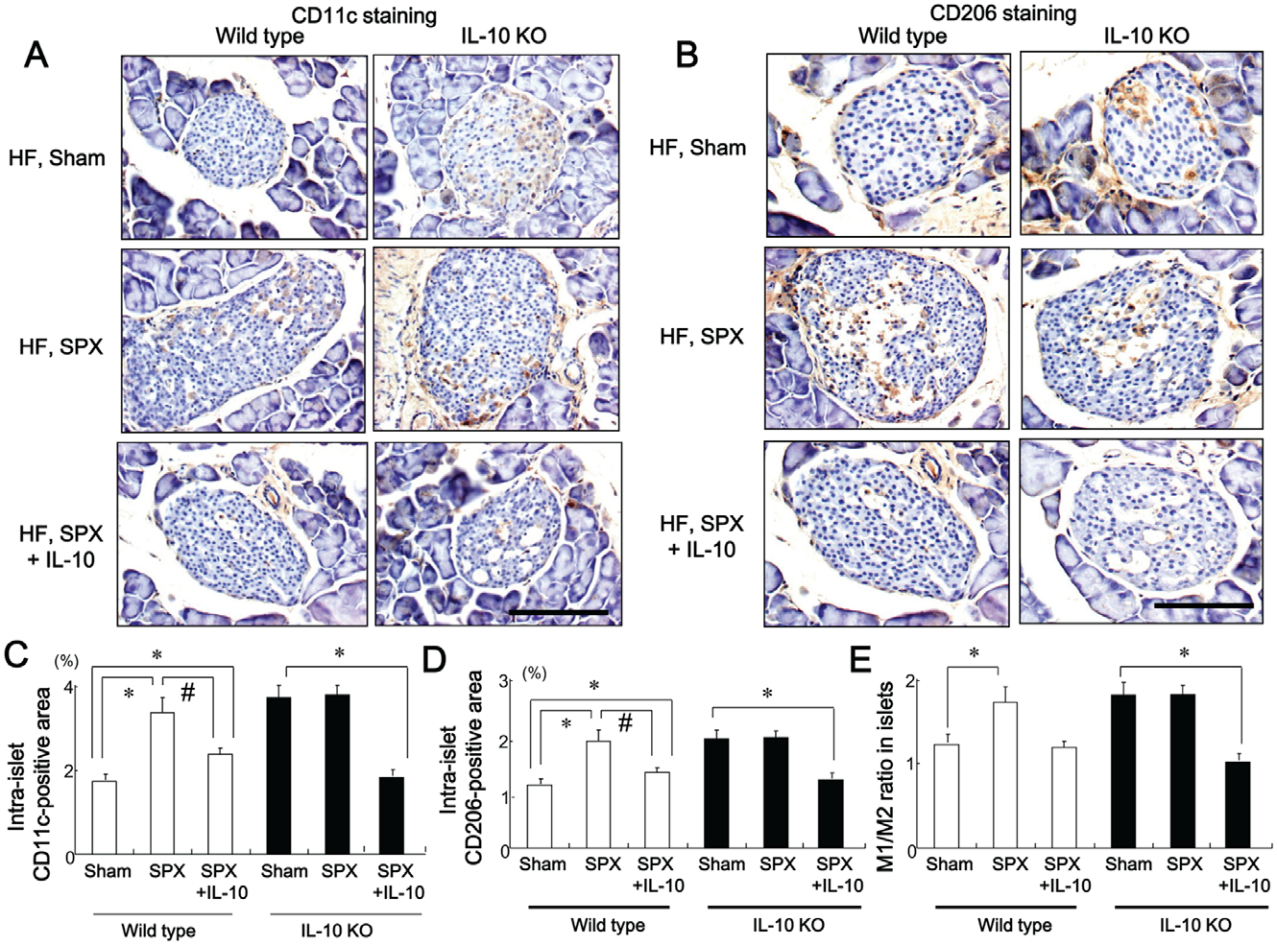
SPX has little effect on the infiltration of macrophages in islets of IL-10-deficient mice. (**A and B**) Representative images of CD11c staining (**A**) and CD206 staining (**B**) in intra-islet areas of pancreas sections from each group. Scale bar = 100 µm. (**C−E**) CD11c-positive areas (**C**), CD206-positive areas (**D**), and M1/M2 ratios (**E**) in the islets in each group (*n* = 6). *P<0.05 vs. the Sham group (wild-type or IL-10KO mice), ^#^ *P*<0.05 vs. SPX mice (wild-type). Treatment groups: Sham, fed a HF diet, given serum albumin, and sham-operated; SPX, fed a HF diet, given serum albumin, and splenectomized; SPX+IL-10, fed a HF diet, given recombinant mouse IL-10, and splenectomized. Wild-type, wild-type mice; IL-10KO, IL-10-deficient mice.

**Figure 9:**
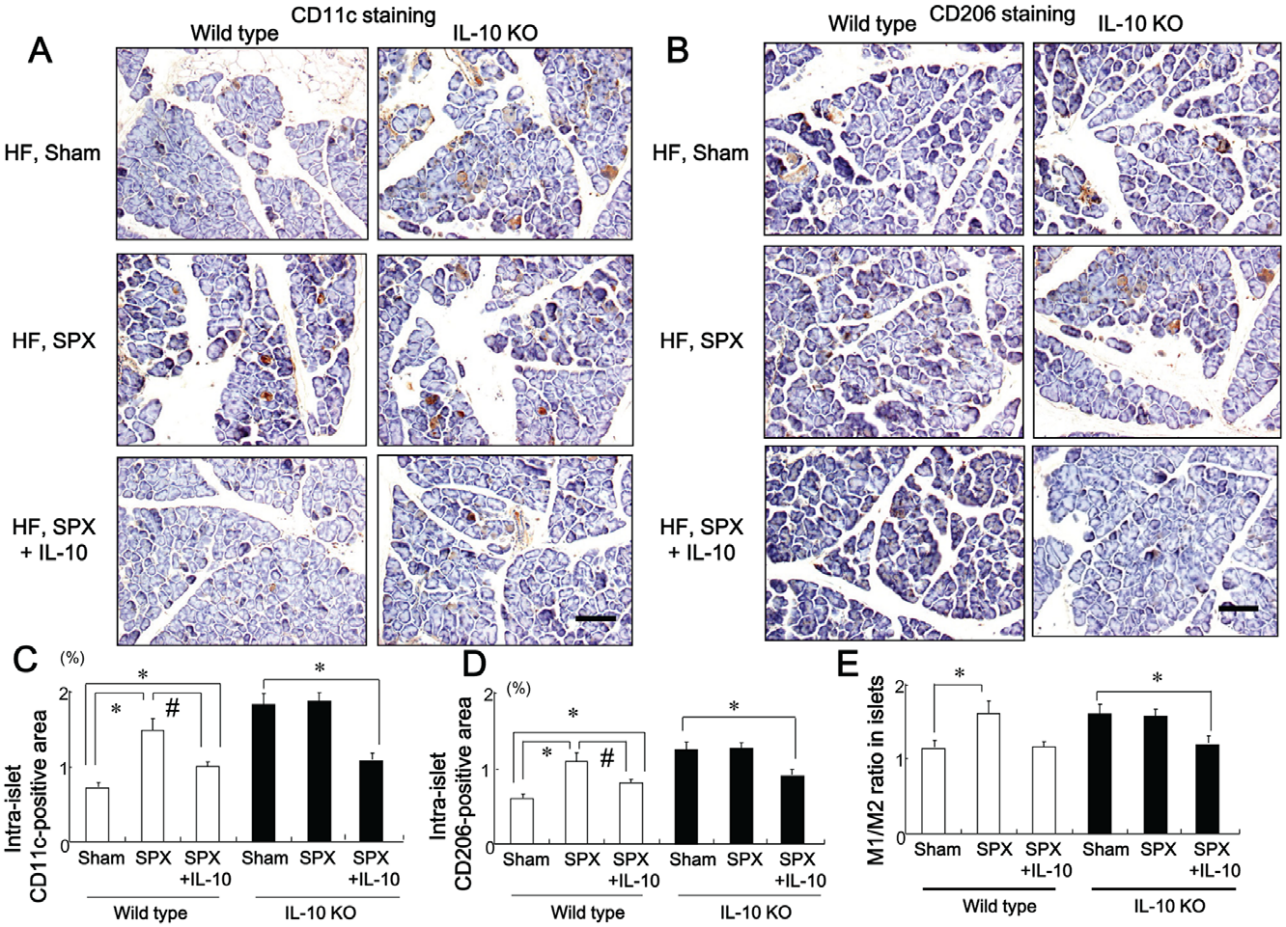
SPX has little effect on the infiltration of macrophages in intra-lobular area of IL-10-deficient mice. (**A and B**) Representative images of CD11c staining (**A**) and CD206 staining (**B**) in intra-lobular areas in pancreas sections from each group. Scale bar = 100 µm. (**C−E**) CD11c-positive areas (**C**), CD206-positive areas (**D**), and M1/M2 ratios (**E**) in intra-lobular areas in each group (*n* = 6). *P<0.05 vs. the Sham group (wild-type or IL-10KO mice), ^#^ *P*<0.05 vs. SPX mice (wild-type). Treatment groups: Sham, fed a HF diet, given serum albumin, and sham-operated; SPX, fed a HF diet, given serum albumin, and splenectomized; SPX+IL-10, fed a HF diet, given recombinant mouse IL-10, and splenectomized. Wild-type, wild-type mice; IL-10KO, IL-10-deficient mice.

**Figure 10:**
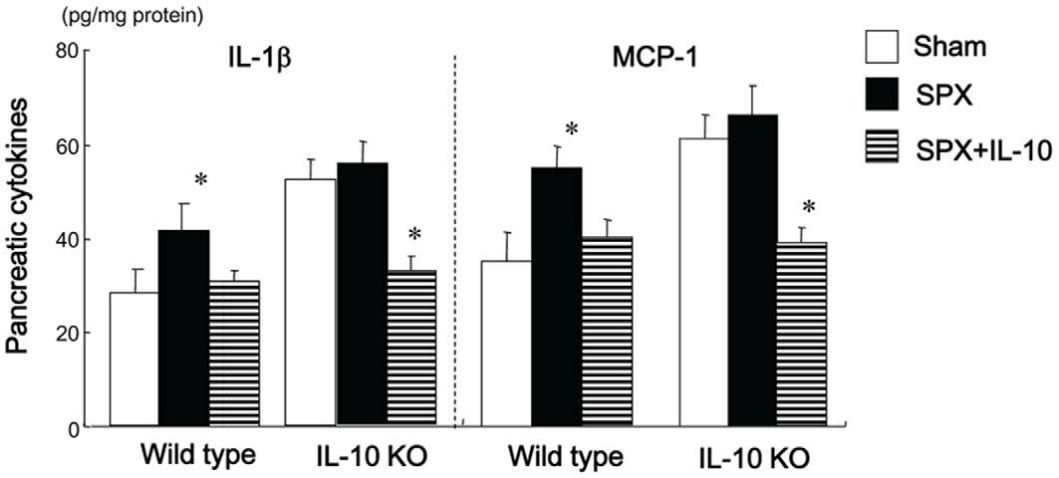
SPX has little effect on pro-inflammatory cytokines in the pancreas of IL-10-deficient mice. Pancreatic content of pro-inflammatory cytokines in each group (*n* = 6). *P<0.05 vs. the Sham group (wild-type or IL-10KO mice). Treatment groups: Sham, fed a HF diet, given serum albumin, and sham-operated; SPX, fed a HF diet, given serum albumin, and splenectomized; SPX+IL-10, fed a HF diet, given recombinant mouse IL-10, and splenectomized. Wild-type, wild-type mice; IL-10KO, IL-10-deficient mice.

We investigated whether SPX altered the insulin-stained area, and intra-islet or intra-lobular fibrosis in the pancreas of IL-10KO mice. The SPX-induced alterations in insulin-stained area (Figure 6A and 6B), intra-islet (Figure 6A and 6C) and intra-lobular areas of fibrosis (Figure 6A and 6D), hydroxyproline content (Figure 6E), number of α-SMA-positive cells (Figure 6A and 6F), accumulation of fat, intra-lobular areas and intra-acinar cell areas of the pancreas (Figure 7A and 7B), pancreatic TG content (Figure 7C), serum leptin level (Figure 7D), infiltration of CD11c-positive (Figure 8A and 8C, 9A and 9C) and CD206-positive cells (Figure 8B and 8D, 9B and 9D) in islets and intra-lobular areas, M1/M2 ratios in intra-islet (Figure 8E) and intra-lobular (Figure 9E) areas, and pancreatic levels of the pro-inflammatory cytokines IL-1β and MCP-1 (Figure 10) that we observed in wild-type mice were not seen in IL-10KO mice. Treatment with IL-10, however, restored these responses in SPX wild-type and IL-10KO mice (Figures 6−10).

## Discussion

Obesity is known to be a low-grade, chronic, pro-inflammatory state [16]−[18] but the primary cause of obesity-induced inflammation is not well understood. To our knowledge, our study is the first to systematically characterize the lack of IL-10 production in the spleen due to HF-induced obesity and to assess the effects of SPX on the pancreas, serum cytokines, and lipid metabolism in mice.

Serum levels of IL-10, but not IL-1β or MCP-1, were significantly lower in HF diet-fed mice compared to mice fed standard chow, despite a significantly decreased expression of all cytokines in the spleens of mice fed the HF diet compared to standard chow-fed controls. Although the spleen is the largest lymphoid organ, it is possible that IL-10 derived from other lymphoid organs, such as mesenteric lymph nodes (MLN), might also be decreased during HF feeding. A previous study reported that obese rats produced higher concentrations of IL-10 in MLN compared to lean rats, but produced approximately 25% lower concentrations of IL-10 in the spleen [19]. For this reason, we focused on spleen-derived IL-10 in the present study. We previously observed that compared to a standard chow, consumption of a HF diet downregulated the expression of CD20, a surface molecule present on B-cells that mainly produce IL-10 in the spleen. These results indicated that consumption of a HF diet reduces the number of B-cells, which play a major role in the immune response, including synthesis of IL-10. Moreover, splenocyte proliferation stimulated by T-cell and B-cell mitogens was reported to be significantly lower in obese subjects and the function of T-cells and B-cells in the spleen was suggested to be impaired in obesity [20]. We therefore hypothesized that the obesity-induced reduction in IL-10 synthesis in the spleen may lead to inflammatory responses in the pancreas and to metabolic disorders.

In the present study, we demonstrated that SPX reduced food intake and body weight, as compared with sham treatment. Furthermore, treatment with IL-10 abolished SPX-induced hypophagia and body weight loss. These findings are consistent with previous data showing that IL-10 administration attenuates inflammation-induced anorexia [21]. We also showed that mice that underwent SPX developed obesity-induced structural alterations in the pancreas, characterized by islet hypertrophy and fibrosis, and ectopic fat deposition (particularly of TGs) in the pancreas that was beyond what would be expected as a result of increased body fat storage. This suggests that NAFPD may result in pancreatic inflammation, since inflammation is known to be closely related to fat accumulation in the pancreas [4]. Furthermore, IL-10 treatment attenuated the SPX-induced alterations in the pancreas. These observations are important because they support our hypothesis that consumption of a HF diet reduces levels of spleen-derived IL-10. In addition, there is experimental evidence to support the notion that HF diet-induced obesity can induce hyperlipidemia and lead to alterations in the pancreas, including increased pancreatic accumulation of fat, and fibrosis caused by activated PSCs [22], [23]. In the present study, increased islet area and pancreatic fibrosis were observed in SPX-treated wild type mice, which were similar to pathologic changes observed in mice fed a HF diet. We previously reported that HF-induced obesity led to reduced synthesis of IL-10 from the spleen, and that systemic administration of IL-10 suppressed SPX-induced insulin resistance and the reduction in serum adiponectin levels [15], [21]. Moreover, the SPX-induced abnormalities in glucose metabolism observed in wild-type-mice were not seen in IL-10KO mice, suggesting that spleen-derived IL-10 is involved in glucose metabolism [15]. Overall, these data are consistent with several studies showing that islet size is increased in obese rodents and that the increase in islet mass results from increased secretory demand resulting from diet-induced obesity and insulin resistance [24], [25]. To this end, in the present study, we observed that SPX accelerated the HF-induced increase in α-SMA-positive cells, which cause fibrosis, and IL-10 treatment suppressed this response. Furthermore, the degree of pancreatic fibrosis was associated with the activation of α-SMA-positive cells and hydroxyproline content in the pancreas. Taken together, these findings indicate that the decline in spleen-derived IL-10 observed with obesity or SPX likely promotes pathologic abnormalities in the pancreas.

To further clarify the ability of spleen-derived IL-10 to protect against ectopic fat accumulation and inflammation induced by SPX, we examined whether IL-10 deficiency affected SPX-induced alterations in the pancreas in IL-10KO mice. Obesity leads to fat infiltration and dysfunction in many visceral organs, including the pancreas [2], [26]. Indeed, a separate study showed that lipotoxicity contributes to the inflammatory response, multisystem organ failure, and necrotic acinar cell death in acute pancreatitis [27]. In the present study, oil-red O staining showed that SPX promoted ectopic fat distribution in intra-lobular areas but not in intra-acinar cell areas, supporting a reported finding of little evidence for fat accumulation within acinar cells in obesity [27]. Furthermore, the SPX-induced fat accumulation that we observed in wild-type mice was not found in IL-10KO mice whereas systemic IL-10 administration decreased this accumulation in both mice. These observations indicate that spleen-derived IL-10 could regulate ectopic fat deposition in the pancreas. Similar to the effects we observed on fat accumulation, SPX-induced pro-inflammatory effects in the pancreas, and islet hypertrophy and fibrosis observed in wild-type mice were not found in IL-10KO mice. Moreover, IL-10 treatment inhibited these alterations in the pancreas in both SPX wild-type and IL-10KO mice, thus supporting the hypothesis that obesity-induced reductions in IL-10 derived from the spleen may result in pancreatic damage. Taken together, these observations suggest that spleen-derived IL-10 may prevent obesity-induced fat accumulation and chronic low-level inflammation in the pancreas. It is also possible that IL-10 deficiency induces basal pancreatic islet inflammation. A previous study demonstrated that absence of IL-10 failed to accelerate spontaneous diabetes but potentiated cyclophosphamide-induced diabetes in non-obese diabetic mice. This finding may be associated with enhanced production of pro-inflammatory cytokines *in vivo*, supporting our observation that HF feeding promoted pancreatic inflammation including lobular and islet areas in IL-10 KO mice compared to wild-type mice [28].

In chronic inflammatory conditions, defective synthesis of IL-10 directly contributes to increased levels of pro-inflammatory cytokines [29]. We determined serum cytokine levels in sham-operated and SPX mice fed standard and HF diets and observed that serum IL-10 levels were reduced by more than half as a result of SPX in mice fed both the standard diet and the HF diet, which induces a strong systemic inflammatory response. Simultaneously, SPX led to elevated expression of IL-10 in the pancreas, thus supporting the assertion that spleen-derived IL-10 has greater effects on systemic inflammation than IL-10 that is produced locally in the pancreas. On the other hand, the decrease in serum pro-inflammatory cytokine levels induced by SPX was at most 40%, suggesting that unlike anti-inflammatory cytokines such as IL-10, pro-inflammatory cytokines are predominantly produced in organs other than the spleen. Indeed, SPX would eliminate production of both pro- and anti-inflammatory cytokines by the spleen. The fact that SPX did not affect fat accumulation or inflammatory responses in the pancreas of IL-10-deficient mice, however, suggests that the SPX-induced alterations that we observed in the pancreas of wild-type mice are not due to the absence of pro-inflammatory cytokines that resulted from SPX.

In this study, SPX altered the expression of pro- and anti-inflammatory cytokines and the infiltration of macrophages into the pancreas, which was accompanied by a decrease in the IL-10/IL-1β ratio. Macrophages, critical components of innate immune responses [30], [31], show significant heterogeneity in function, since their properties and activation state are shaped by local environmental factors [30], [32]. Macrophage activation has been operationally defined across two separate polarization states: M1 or “classically activated” and M2 or “alternatively activated” macrophages [30], [32]. M2 macrophages produce high levels of anti-inflammatory cytokines such as IL-10 and low levels of pro-inflammatory cytokines [33]. To investigate the presence of activated PSCs in the pancreas, we performed immunohistochemical staining of pancreatic tissue sections with α-SMA and observed that the alteration of IL-10 content in the pancreas paralleled changes in the number of α-SMA-positive cells in each group. Interestingly, it has been reported that that IL-4-transducted PSCs synthesize IL-10 significantly [34]. These findings indicate that the main sources of pancreatic IL-10 are activated M2 macrophages and/or PSCs. It is generally accepted that consumption of a HF diet triggers the recruitment of M1 macrophages, which release pro-inflammatory cytokines, thereby causing metabolic disorders [30]. It can therefore be assumed from the present findings that conditions favoring the pro-inflammatory M1 polarization of macrophages, such as reduction of splenic IL-10 release in obesity, play a role in the development of ectopic fat accumulation and fibrosis due to inflammation.

Macrophage polarization is generally considered a conceptual framework for a continuum of functional states [35]. A previous study indicated that macrophage polarization might be viewed as more skewed toward the M1 phenotype during coxsackievirus (CVB)4-V infection, which progresses to chronic pancreatitis and toward the M2 phenotype during CVB4-P infection, which induces acute, interstitial pancreatitis [36]. To characterize the impact of spleen-derived IL-10 on macrophage polarization in obesity-related pancreatic damage, we analyzed the pancreatic expression of a panel of M1 and M2 macrophage markers in the present study. The expression level of both M1 and M2 macrophages, as well as the M1/M2 ratio, were elevated in the pancreas of HF diet-fed mice compared to standard chow-fed mice. In addition, these responses were further amplified by SPX in wild-type, but not IL-10KO mice, and supplementation of IL-10 in SPX wild-type and IL-10KO mice was associated with downregulation of M1 markers and the M1/M2 ratio, along with upregulation of M2 markers in the pancreas. These data suggest that the lack of spleen-derived IL-10 may contribute to the abnormal activation of the inflammatory state in the pancreas. The mechanism underlying the alterations in M1 and M2 macrophage numbers in the pancreas is unclear, but one possible explanation is that spleen-derived IL-10 is involved in the regulation of the M1/M2 balance in local tissues.

In humans, circulating leptin concentration increases directly with increasing obesity. Higher circulating concentration of leptin, a proinflammatory molecule, is associated with many autoimmune and inflammatory diseases, such as inflammatory bowel disease [37]. The role of leptin in pancreatitis, however, is unclear. One study reported that in pancreatitis induced by cerulean injection in lean rats, serum leptin concentration was increased [38]. Following induction of a disease of greater severity, leptin concentrations were high at 12 and 24 h but similar at 48 h [38]. In the present study, SPX decreased serum leptin levels in wild-type but not IL-10KO mice and IL-10 administration restored serum leptin levels in both mice. These findings are compatible with our previous studies showing that SPX induced loss of body weight due to decreased food intake and increased oxygen consumption but accelerated inflammation in visceral fat while IL-10 treatment increased body weight and suppressed visceral fat inflammation [15], [21]. Although the serum concentration of leptin might increase in pancreatitis, there seems to be no relationship between severity of the disease and circulating leptin concentration.

In conclusion, we have demonstrated a critical role for spleen-derived IL-10 in pancreatic inflammation in the obese state. Although additional work is needed to understand the mechanism by which obesity elicits an inflammatory response, the results of this study contribute a more comprehensive understanding of the interactions between obesity and the spleen and may facilitate the development of therapeutic strategies for treating NAFPD.
